# Bowlby's five therapeutic tasks: bringing them up to date for children

**DOI:** 10.1192/bjb.2023.67

**Published:** 2024-08

**Authors:** Simon R. Wilkinson

**Affiliations:** Retired, Oslo, Norway

**Keywords:** Secure base, psychotherapy, child psychiatry, family therapy, carers

## Abstract

Bowlby remained attached to his psychoanalytic roots and conceptualised treatment in terms of one-to-one relationships, albeit acknowledging the need for a family formulation. Bowlby's five therapeutic tasks were never adapted to the current understanding of working with the relationships fostering the development and maintenance of children's attachment strategies. This paper goes through each of Bowlby's five tasks and adapts them to our current understanding of development, with consequences for prioritising family approaches, rather than a secure base alone with a therapist. In doing so I will review the process of achieving security, seeing it as more similar to an allostatic process than a state of homeostasis.

The initial developments of attachment theory following the work of Bowlby have been summarised by Duschinsky.^1^ There have been various attempts to classify and categorise attachment behaviours and their subsequent strategic applications in handling relationships. Associated with these attempts, there are various ways of measuring ‘attachment’. Attachment has been reified as something that one has and keeps (the ‘bond’ school) – and also as something that is dimensional, can change and is hard to quantify – in effect, is not truly measurable – as it shows its colours in relationships (the ‘adaptation’ school). This paper does not explore these various schools of thought and their diverse applications of attachment measures or approaches to therapy. Instead it aims to review the premises of Bowlby's suggested five tasks of treatment through the eyes of the current understanding of the predictive brain hypothesis^2^ and the functioning of memory systems. This has the effect of allying my approach most closely with the adaptation school associated with the dynamic maturational model of attachment and adaptation (DMM).^3,4^

## Bowlby's five therapeutic tasks: the primary need for security

Bowlby's break with the paradigms then dominant in the British Psychoanalytical Society centred around the role of trauma in understanding treatment needs. He, along with colleagues in what became known as the Independent Group of British analysts, disagreed with the premise that the trauma was in fantasy, believing it was real. This put security in the forefront of what was to become attachment theory.

Bowlby's five tasks (^5^, pp. 138–139) are presented as necessary for treatment of adults in psychotherapy, where addressing the first is a prerequisite for addressing the other tasks. He focuses on the role of the therapist. Here are abridged versions of the original five:

1. ‘provide the patient with a secure base [ … ] with a trusted companion to provide support, encouragement, sympathy and, on occasion, guidance’
2. ‘encourage him to consider the ways in which he engages in relationships with significant figures in his current life, what his expectations are for his own feelings and behaviour and for those of other people’
3. ‘encourage the patient to examine [ … ] the relationship between the two of them’, i.e. the therapist with patient
4. ‘encourage the patient to consider how his current perceptions and expectations and the feelings and actions to which they give rise may be the product either of the events and situations he encountered [ …. ] especially those with his parents, or else as the products of what he may have repeatedly been told by them’
5. ‘enable the patient to recognize that his images (models) of himself and of others [ … ] may or may not be appropriate to his present and future’.

When applying attachment theory to inform therapy, the idea of a ‘secure base’ has been given prime importance, seen by Bowlby as the most important attribute provided by a therapist for an individual patient (^5^, p. 140). It has been transferred to the clinical world of child psychiatry without question. I suggest that the premise in child psychiatry is to ensure that the child's *home* is safe *enough* for therapy to proceed and that this is best examined and facilitated through working, as soon as possible, directly with those living together as one unit. The home is to become the secure enough base rather than prioritising the relationship with the therapist. The therapist's job is to contain enough of the tension that can otherwise disrupt the therapeutic process with the family members, the ‘holding’ function as described by Winnicott. But the therapist must not forget the need to increase tension when appropriate, as retrieval from emotional memory is facilitated when the tension is more similar to the state under which the memory was laid down (^6^, pp. 13–14). Undesirable emotional memories can be actively suppressed, initially dependent on a prefrontal process.^7^

Security is not something that one has or doesn't have. It has to be pursued continuously. The world is a risky place with the potential for danger always present, unfortunately also from therapists. We are all continually working out what behaviour is necessary on our part to maximise our chances of tackling whatever the day, or night, might throw at us. Security reflects an allostatic process to manage a threat/danger stress load. This active process requires a flexibility and a wide repertoire of ways of behaving. It requires that we read the signals available to us about what might be in store. Understanding forms of deception is necessary for this process, especially picking up paradoxes in the information available. Our ability to deceive increases with age and experience of need to do so.

To anticipate what might be required of us we rely on our predictive brain.^2^ We organise the information coming to us, both exteroceptive and interoceptive. We filter it (not to be understood as a conscious process) to allow the most relevant information for managing danger/threat access to higher levels of integration with the activity patterns continuously being generated and updated to guide our responses. Bateson's concept of information, in terms of the difference that makes a difference (^8^, p. 286), finds its current equivalent in the processes hypothesised to guide predictive brain functioning. The activity patterns preparing us for relevant action are being continuously reformulated better to fit the important information making a difference to us, which has filtered higher up in our brain. Past experience is meeting the current incoming information and a best possible suitable ‘survival’/‘comfort’ strategy is made available for us. The past is being put to service to maintain our security and sense of affective balance. Attachment theory has become an information processing theory,^3^ enabling our adaptation to our environmental niche, and the processes therein governing what is likely to be required of us in relation to other people.

It will always be useful to see or sense more danger than is actually present – just not too much more! When sensing danger, we need to act first and not use too much time to think about what to do to be safe. The time for reflection comes later. Therefore our memory systems play a key role in prioritising our implicit learning when action has to come first – our procedural memory providing the template for action in relation to the perceptuo-affective information being responded to. We respond predominantly to each other's behaviour, even though we might rather say things more consistent with what was said. Verbal language provides the more effective route to deception. This was very clear to me once in a party political broadcast in the UK when the leading politician's head movements signalled ‘no’, but the words that accompanied said ‘yes’. The interviewer responded as if yes was true, whereas any infant would know that they should respond to the ‘no’.

Seeing security as a dynamic allostatic process, one involving both interoceptive and exteroceptive information continuously changing in real time, leads to a need to understand how we come to attribute meaning to what we see and experience. The greater the anticipated danger the more impulsive the response. Understanding implicit learning puts us on a different footing in therapy, as what we talk about plays second fiddle. Our somatic experiences are gate-crashing our cognitive processes below the level of awareness, and, as we are being behaviourally adapted to learning the short cuts necessary for survival and for achieving affective balance, this gate-crashing is enabling that feeling of being on top of things (see Kozlowska^9^ for elaboration of the range of somatic responses under duress and the roles they can play in misunderstanding each other while maintaining a degree of family cohesion). Our implicit learning is preconscious and depends on behavioural learning principles. Through working with the contingencies that maintain the priority being given to such information there is the greatest chance for change. This necessitates working directly with the contingencies in the family response patterns. Such work with the family members addresses maintaining factors – which is not the same as saying these were the precipitating dynamics.

Therapy has usually concentrated on processes connected to explicit memories, both semantic and, even more importantly, episodic for recall of events. But we know that false memories and reports of things that have reputedly happened are rife (^6^, pp. 33–37). Episodic memory is unreliable,^10^ especially concerning trauma, as such information easily falls prey to the ways in which information can be transformed (^11^, pp. 47–55). Our child patients’ VIPs (I prefer the openness of ‘very important people’ to cover all major caregivers, rather than using parents, mothers, etc.) are trying to read their children's signals as helpfully as possible, without emotionally overloading their children. Their spyglasses exaggerate some signs to the detriment of others, depending on their own life histories. Their emotional responses to the signs can be out of control if their implicit learning dominates. Of necessity, these will introduce biases in how their children monitor their own states and how they identify when they believe themselves to be seriously ‘out-of-sorts’. They come to imply that action is needed, rather than the state of discomfort being something that can be lived with.

To set up an optimal secure enough therapy situation it becomes necessary to address the dynamic between VIP and child directly. The ‘family’ (used to cover all units catering for the developmental needs of children – including children's homes, etc.) becomes the place where maintaining factors may be inadvertently fostering the child's dis-ease or dis-stress (the usual word is stress, but we are now more aware of the potential beneficial effects of stress – termed eustress – and I use dis-stress to convey the negative quality of duress). The family members have their own histories of trauma, and danger, both real and imagined. The ‘five tasks’ need to start with the dynamic processes required to maximise enough security in the family for treatment to proceed. This might concern current events or past events biasing interpreting the dangers inherent in the child's world. This cannot be achieved through seeing the child alone, and it is not sufficient to have parallel sessions with the VIPs. The actual interactions within the family need to be observed and acted upon, rather than limiting oneself to what is reported. My thesis is that safety in the family unit needs to be addressed first so that the family becomes the familiar ‘safe enough base’ – rather than the one-to-one relationship with a therapist functioning as if that was the ‘secure base’. This leads to the revised level-one task in the list that follows. This is the critical step and requires comprehensive work.

## An alternative version of the tasks

For ease of comparison with the original list I itemise all my suggested modified five tasks.

1. Ensure that the home situation is safe enough for everyone in the family. No family members are currently in danger or under threat. Together they have enough will and resources to support and encourage each other.
2. Encourage everyone in the family to consider the ways in which they engage with each other when under duress, what their expectations are of each other and how they resolve their needs of each other. Note the recurring patterns and care available. Especially encourage everyone to explore what their expectations are of the referred patient's symptoms and how the symptoms function in transactions between the family members.
3. What is each family member's understanding of the symptoms, given their own experience of similar symptoms? Encourage the family to consider how their current perceptions and expectations, and the feelings and actions to which they give rise, may be the product either of events and situations encountered or of what they may have repeatedly been told. It might help to know how the grandparents responded to similar symptoms in the parents or whether such symptoms would have been impossible for the grandparents to acknowledge.
4. Develop the above through exploring how the symptoms pattern the family's relationship with the health services or other caring professions. Is the same pattern being repeated with other professions involved with the patient? Are any of the involved professionals seen as indispensable for keeping the family in harmony – and, if so, does this repeat a pattern within the family?
5. Everyone in the family re-evaluates their own role in responding to symptomatic behaviour and how this may or may not be appropriate now or in the future.

The concluding elements in the first task – will, support and mutual encouragement – reflect my transfer of elements in Bowlby's first task to the family. Yet these elements are difficult to achieve and necessitate integrating the subsequent tasks in the process towards achieving *enough* security in the family. The tasks recursively enrich each other.

To get started on this all important first goal of achieving enough security in the family, we need a strategy to form an alliance with each member of the family. This becomes the fundamental step from which we proceed.

### Treatment alliance

We engage with the family members through two different access points. They have been referred because of distress of some kind. It might be the VIPs’ concerning their child, or in the child. The child's distress may be clearly communicated or silently managed, accurately described or misleading. How do the symptoms function within the family and illuminate the dynamic with the VIPs and patient under duress? What have the VIPs been aware of and how does that mesh with their own histories or knowledge about what they believe might be the matter? This will set the parameters for how poorly they consider their child to be and the responses expected of them and put in motion a family dynamic in which the various caregivers may or not agree. The way in which they handle their different perspectives leads to another level of complexity in the dynamic that arises. Sociologists might term this the sickness dimension, the state that the caregivers attribute to the child. This contrasts with the illness dimension of subjective distress being experienced by the child (^12^, pp. 37–38). It is the latter that forms the foundation on which the alliance with the patient can be built. The alliance with the VIPs depends on acknowledging what they have observed and the values they attribute to their observations. The alliance with the child depends on acknowledging her subjective discomfort, in the degree she chooses to share it, or acknowledging her physical complaints.

There are also hidden partners in the alliance to be built: the absent interested parties of both the referring person (see for example Palazzoli et al^13^) and the economic drivers of approved practice associated with the diagnostic system being used in the particular health service. These will not be addressed here. I only hope they will not be ignored.

### The recursive relation between the tasks

Change involves taking a risk when doing something differently. Our predictive brain will evaluate what sort of risk is involved so that we do not take too big a chance and put ourselves in danger. It necessitates that our implicit memory does not reflexively dominate our response, that there is enough time to reflect on a wider range of signals being evaluated, which can give us an opening to do something new. This is one variety of where thinking fast needs to give way to thinking slow enough to reflect on the potentials of the situation. Once all members of the family feel secure enough to take chances, therapy advances rapidly. The challenge is to get there, so that new learning regularly takes precedence over previous learning even when there is a whiff of danger. Eventually, repeated practice and minimising the triggers for return to previous practice enable durable change.

Montalvo & Haley^14^ described the way in which seeing the child alone could work through making the parents feel more able to relax and allow themselves to see their child in a new light. The non-judgemental approach gives the VIPs room to change. This is seen as a form of family therapy, with the therapy being an attitude to the problem, not a method. It may be that although Bowlby did not invest in family therapy as such, his emphasis on a family formulation is in keeping with the attitude described by these authors. His approach had been to meet with the family members together before treatment proceeded, once with each of the parents alone and the child alone.^15^

Establishing the attitude required necessitates employing points 2–5 of the modified tasks above. They can be sewn together within the framework of the alliance-building elements. Each point is recursively linked to moving the level of security from marginally good enough to that capable of fostering further change without therapist involvement. During this process the therapist helps the family to grow into as secure a family base as possible, through being non-judgemental and modulating the level of tension up or down when necessary.

If the family cannot reach marginal security it may be that a couples approach or individual work with the adults is required before the children are included. Marginal security necessitates that the VIPs are willing to support therapy and become actively involved themselves. The basic requirements for daily living can be met, so that there is enough food, shelter, warmth and the possibility for sleep. If these cannot be met other agencies need to be involved.

If work with the VIPs alone has to take precedence, this is not because of a criticism of their parenting ability in the past, but an acknowledgement that in the current situation their own pasts appear to trigger impulsive responses that are maintaining the dis-stress in the child. In their current context they appear unable to modify these responses. Their own reflective functioning needs to advance before the first task can be addressed. Their willingness to be involved is not enough on its own. This is especially problematic for the children of parents with high levels of expressed emotion, as such parents have greater tendencies to blame the children and so are unavailable to provide the support and encouragement required within the family.^16,17^

The circle of security approach,^18^ which helps VIPs develop competence in reading the signals of their children, can function as one step to increasing the chance of safety at home.^19^ It is not primarily aimed at parents with the greatest challenges. It only indirectly addresses the implicit driving force from the VIPs’ earlier learning, maintaining impulsive actions without reflection, potentially to the detriment of the advantages to be had from being able to read their children's signs more helpfully. The unpredictability of an impulsive response can threaten the security of the otherwise growing sense of safety at home. Parental unpredictability, especially intensely expressed emotion, can be expected to trump the gains from being able to read their children's emotional language.

## An ingredient Bowlby left out

Although my main interest has been to reflect on the five psychotherapeutic tasks in the light of current developments in developmental psychopathology and attachment theory, society's conceptualisation of mental disorder as concerning DSM- or ICD-approved diagnoses means another ingredient has to be added to any treatment plan. In shorthand, the Bowlby tasks can be said to develop reflective function in the service of change. But the role played by developmental vulnerabilities has been left unaddressed. If we were to design a more comprehensive set of tasks they would have to include identification of such vulnerabilities, be these genetic, birth injuries or the consequences of divergent early brain development, among others (I elaborate on the focus for treatment in child psychiatry in an another paper^20^).

## Conclusions

I suggest that Bowlby's formulation of five therapeutic tasks needs revising to facilitate the development of therapy in child psychiatry. A secure base for a child alone with a therapist is not enough. Individual approaches to child patients should not emphasise a secure relationship to an individual therapist at the cost of formulating the treatment plan in terms of facilitating primarily the development of a secure enough family base. Therapy is a cybernetic systems approach whereby the inherent dangers in life cease impulsively to govern us, and instead can be reflected on and inform the humanity of man to man.

## Data Availability

Data availability is not applicable to this article as no new data were created or analysed in this study.

## About the author

**Simon R. Wilkinson**, MD, FRCPsych, is a retired consultant child and adolescent psychiatrist, now settled in Oslo, Norway.
